# Mercury concentrations and distribution in soil, water, mine waste leachates, and air in and around mercury mines in the Big Bend region, Texas, USA

**DOI:** 10.1007/s10653-014-9628-1

**Published:** 2014-06-29

**Authors:** John E. Gray, Peter M. Theodorakos, David L. Fey, David P. Krabbenhoft

**Affiliations:** 1 U.S. Geological Survey, MS 973, Federal Center, Denver, CO 80225 USA; 2 U.S. Geological Survey, 8505 Research Way, Middleton, WI 53562 USA

**Keywords:** Mercury, Soil, Stream water, Air, Leachates

## Abstract

Samples of soil, water, mine waste leachates, soil gas, and air were collected from areas mined for mercury (Hg) and baseline sites in the Big Bend area, Texas, to evaluate potential Hg contamination in the region. Soil samples collected within 300 m of an inactive Hg mine contained elevated Hg concentrations (3.8–11 µg/g), which were considerably higher than Hg in soil collected from baseline sites (0.03–0.05 µg/g) distal (as much as 24 km) from mines. Only three soil samples collected within 300 m of the mine exceeded the probable effect concentration for Hg of 1.06 µg/g, above which harmful effects are likely to be observed in sediment-dwelling organisms. Concentrations of Hg in mine water runoff (7.9–14 ng/L) were generally higher than those found in springs and wells (0.05–3.1 ng/L), baseline streams (1.1–9.7 ng/L), and sources of drinking water (0.63–9.1 ng/L) collected in the Big Bend region. Concentrations of Hg in all water samples collected in this study were considerably below the 2,000 ng/L drinking water Hg guideline and the 770 ng/L guideline recommended by the U.S. Environmental Protection Agency (USEPA) to protect aquatic wildlife from chronic effects of Hg. Concentrations of Hg in water leachates obtained from leaching of mine wastes varied widely from <0.001 to 760 µg of Hg in leachate/g of sample leached, but only one leachate exceeded the USEPA Hg industrial soil screening level of 31 µg/g. Concentrations of Hg in soil gas collected at mined sites (690–82,000 ng/m^3^) were highly elevated compared to soil gas collected from baseline sites (1.2–77 ng/m^3^). However, air collected from mined areas at a height of 2 m above the ground surface contained concentrations of Hg (4.9–64 ng/m^3^) that were considerably lower than Hg in soil gas from the mined areas. Although concentrations of Hg emitted from mine-contaminated soils and mine wastes were elevated, persistent wind in southwest Texas disperses Hg in the air within a few meters of the ground surface.

## Introduction

Contamination of land, water, and air by Hg has been known as a global issue for many years (WHO 1976; Nriagu and Pacyna 1988; Fitzgerald and Clarkson 1991). Mercury has no biological function in humans and exposure to high Hg concentrations is a potential hazard (NAS 1978; Eisler 1987; USEPA 1997). All forms of Hg are toxic, but organic Hg compounds such as methyl-Hg (CH_3_Hg^+^) are the most toxic (WHO 1990; Ullrich et al. 2001). Inorganic Hg in land sources is potentially converted to methyl-Hg in aquatic environments by the action of microorganisms, especially in organic-rich sediment (Compeau and Bartha 1985).

Areas previously mined for Hg are of potential concern as Hg can be extremely high in mine waste calcine (retorted ore) and runoff sediment, exceeding 1 % by weight in some cases (Gray et al. 2002, 2004). Concentrations of Hg remain highly elevated in soil, sediment, and water near mined areas many years after the cessation of Hg mining (Gosar et al. 1997; Bailey et al. 2002; Qiu et al. 2005; Gray and Hines 2006; Rimondi et al. 2012). Similarly, Hg concentrations are highly elevated around mines of the Terlingua district located in and around Big Bend National Park (BBNP) (Gray et al. 2006, 2008). Mining of Hg in the Big Bend region, Texas, was carried out from 1888 to 1973 and constituted an important Hg resource in the USA. Production of Hg exceeded 5,000 t from mines of the Terlingua district, ranking it as the third largest Hg district in the USA. Like most Hg mines worldwide, the dominant Hg ore in the Terlingua district was cinnabar (hexagonal, HgS), but metacinnabar (isometric, HgS), elemental Hg (${\text{Hg}}_{{({\text{L}})}}^{0}$), and Hg chlorides such as calomel (Hg_2_Cl_2_), and oxychlorides such as terlinguaite (Hg_2_ClO) and eglestonite (Hg_2_Cl_2_O) were identified at some mines (Ross 1941). A considerable volume of mine waste calcine generated during mining is present at mines in this region (Gray et al. 2006). These calcines contain highly elevated concentrations of Hg, which are available for downstream and downwind transport, leaching, and biogeochemical transformation to methyl-Hg.

**Fig. 1:**
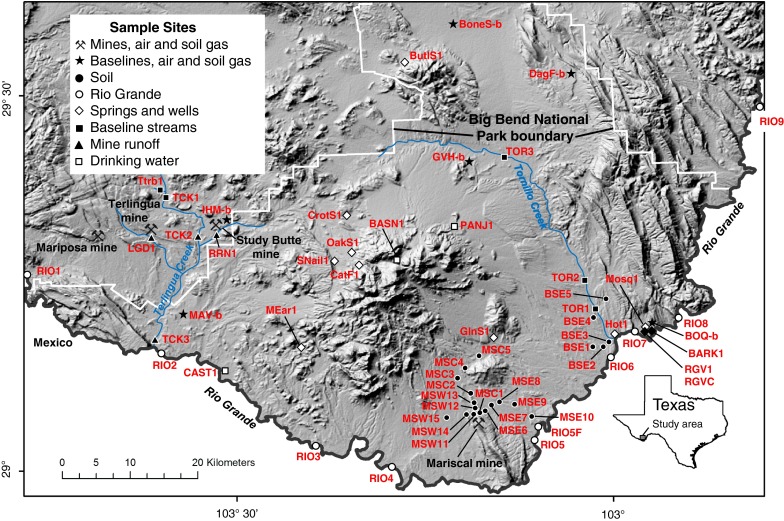
Study area and location of sample sites

The objective of this study was to evaluate potential Hg contamination of soil, water, and air in the Big Bend region. With the exception of the Mariscal mine, mines of the Terlingua Hg district are located just outside of the BBNP boundary (Fig. 1). To evaluate potential Hg contamination in this region, concentrations of Hg and/or methyl-Hg were measured in (1) soil collected proximal to the Mariscal mine in BBNP, (2) stream water collected proximal and distal from mined areas, (3) water leachates obtained from leaching of mine waste calcine, and (4) soil gas and air collected from mined areas and baseline sites distal from mines.

### Study area

The area of study was the Terlingua Hg district, southwest Texas. Mining of Hg was carried out in this region between about 1888 and 1973 (Sharp 1980; Avery et al. 1996) and total production of Hg was >5,000 t, which ranks Terlingua as the third largest Hg district in the USA. One consequence of Hg mining is that considerable mine waste is generated during ore processing, which is typically discarded on the mine site, and much of this mine waste contains toxic Hg compounds. Greater than 2,000,000 m^3^ of mine waste, calcine remains in the Terlingua district and about 30,000 m^3^ at the Mariscal mine. This mine waste contains elevated Hg concentrations due to incomplete Hg recovery and generation of Hg byproduct compounds during retorting, some of which are water soluble (Kim et al. 2003; Gray et al. 2010).

The study area is in the Chihuahuan Desert. During fieldwork, the climate was generally hot (26–41 °C), dry, and sunny to partly cloudy. The wind direction is dominantly from the south and southwest in this region. Precipitation in this area averages about 25 cm/year, occurring principally as storm events. Generally, most precipitation in this region falls from July to September, but this is temporally and spatially variable. Most of the streams and smaller tributaries in the study area are ephemeral, and these streams were sampled during or shortly after storm events. However, the Rio Grande is perennial.

## Methods

### Sample collection and preparation

#### Soil

Soil samples were collected from 15 sites in the Mariscal mine area, and an additional five samples were collected from baseline sites distal from Hg mines in the area (Fig. 1, Table 1). The soil samples were collected in amber glass vessels with Teflon-lined lids and were frozen until analysis. Soil was collected from the near-surface A-horizon at depths of about 1–5 cm following the removal of any rock fragments. The sites generally contained low amounts of organic matter, and no O-horizon was present at any of the collection sites. These soil samples contained fine-grained material consisting dominantly of fine silt and clay-sized material. Soil collected from the three sites most proximal to the Mariscal mine (Table 1, MSC1, MSE6, and MSW11) contained some fine-grained mine waste calcine, which was likely windblown from calcine piles found in this mined area. Prior to geochemical analysis, the soil samples were air-dried at room temperature and then pulverized in a ceramic plate grinder to a grain size of <150 µm.

**Table 1:** Geochemical data for soil samples

Sample #	Location	Hg μg/g	Methyl-Hg ng/g	Methyl-Hg/Hg %	TOC %
*North traverse*					
MSC1	30 m N of Mariscal mine	11	0.54	0.005	0.68
MSC2	800 m N of Mariscal mine	0.07	0.09	0.12	0.38
MSC3	1.5 km N of Mariscal mine	0.02	0.10	0.44	0.37
MSC4	4.5 km N of Mariscal mine	0.03	0.16	0.61	0.30
MSC5	6 km N of Mariscal mine	0.14	0.09	0.06	0.33
*East traverse*					
MSE6	300 m E of Mariscal mine	3.8	0.51	0.01	0.54
MSE7	750 m E of Mariscal mine	0.81	0.15	0.02	0.47
MSE8	2.5 km E of Mariscal mine	0.04	0.05	0.13	0.30
MSE9	5 km E of Mariscal mine	0.05	0.07	0.14	0.51
MSE10	7.5 km E of Mariscal mine	0.05	0.07	0.14	0.28
*West traverse*					
MSW11	100 m N of Mariscal mine	6.2	0.43	0.007	0.82
MSW12	400 m N of Mariscal mine	0.04	0.04	0.10	0.49
MSW13	600 m N of Mariscal mine	0.57	0.63	0.11	1.0
MSW14	1 km W of Mariscal mine	0.18	0.29	0.16	0.24
MSW15	3 km W of Mariscal mine	0.04	0.01	0.03	0.32
*Baselines*					
BSE1	18 km E of Mariscal mine	0.03	0.03	0.10	0.17
BSE2	19 km E of Mariscal mine	0.03	0.02	0.05	0.35
BSE3	20 km E of Mariscal mine	0.03	0.04	0.17	0.34
BSE4	21 km NE of Mariscal mine	0.03	0.03	0.10	0.45
BSE5	24 km NE of Mariscal mine	0.05	0.03	0.06	0.50

Sample numbers correspond to those shown in Fig. 1

#### Mine waste calcine

Samples of mine waste calcine used for the leachate studies were those from an archived collection that were used in a previously published study (Gray et al. 2006). Grab samples of calcine were collected about 25–50 cm below the waste-pile surface to avoid the highly oxidized, near-surface environment and sieved to −2 mm in the field. Calcine is retorted ore that is composed dominantly of rock fragments and minerals such as quartz, feldspar, clay, hematite, and mica. Calcine also contains some cinnabar that survives retorting as well as elemental Hg and Hg minerals such as chlorides and oxychlorides that are formed during retorting, which generally constitute <1 % of the calcine material (Gray et al. 2010). Calcine samples were frozen, and prior to geochemical analysis, they were air-dried and then pulverized in a ceramic plate grinder to a grain size of <150 µm.

#### Leachates

Mine waste calcine samples were leached using a modified version of the USEPA synthetic precipitation leaching procedure, Method 1312 (USEPA 1994), to evaluate the capacity of these sample to leach Hg when exposed to water in a laboratory setting. Following the 1312 Method, 100 g of sample was leached with 2 L of deionized water acidified to a pH of 4.2, and the samples were then rotated at 28 rpm for 18 h. Our only modification of the 1312 Method was that the leachate was collected as four separate samples including an unfiltered leachate sample, and leachate water filtered at 0.70, 0.45, and 0.20 µm, rather than only a 0.70-µm filtered sample as typically used in the 1312 Method. These four separate leachate samples were collected to evaluate if Hg is predominantly in a particulate form after leaching.

#### Water

Samples of unfiltered water were collected from surface streams, the Rio Grande, several wells and springs, and drinking water sources in and around BBNP (Fig. 1). Water samples for Hg and methyl-Hg analyses were collected in Teflon bottles pre-cleaned by boiling in concentrated HNO_3_ for 48 h. The water samples were acidified on site with Baker Instra-analyzed^®^ HCl using a final acid concentration of 0.5 % v/v. Water samples were also collected for dissolved organic carbon (DOC) determinations in amber glass vessels. These amber bottles were pre-cleaned by baking in a muffle furnace at 450 C for 4 h. The DOC water samples were filtered through C-free 0.7-µm borosilicate glass filters into the glass vessels and immediately acidified with ultra-pure HCl to a final acid concentration of 0.5 % v/v.

#### Soil gas and air

Collection of Hg from air and soil gas was carried out using methods similar to those previously described (Gabriel et al. 2005; Walvoord et al. 2008). Collection of Hg from air was made using borosilicate glass columns packed with Au-coated glass beads attached to a battery-operated pump in which air was drawn through the Au trap at a rate of 2 L/min for 20 min. The Au trap was attached to plastic rod at a height of 2 m above the ground surface. The collection line also contained a soda lime/quartz wool trap to eliminate humidity and dirt particulates from entering the Au trap. All Au traps used in this study were pre-cleaned in a controlled laboratory setting by heating to 350 °C under a constant stream of Hg-free Ar, and then, each trap was sealed using Teflon stoppers and then double-bagged in zipped plastic bags.

**Fig. 2:**
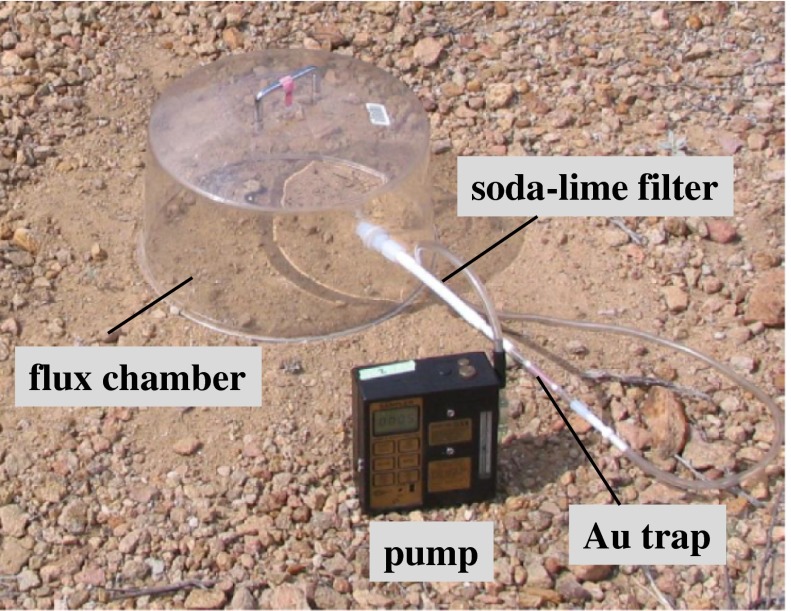
Photograph of soil gas collection. Soil gas is pumped from the flux chamber, through a plastic line with a soda lime/quartz wool filter, and Hg vapor is collected on a Au trap

Collection of Hg from soil gas also was made using Au traps with soda lime traps that were attached to a flux chamber placed directly on a soil or mine waste surface (Fig. 2), and the soil gas was drawn through the trap at 2 L/min for set times, generally 20 min at baseline sites and 5 min at mine sites. After each sample collection, the Au traps were re-plugged with a Teflon stopper, wrapped with Teflon tape, double-bagged in the zipped plastic bags, and sent to the U.S. Geological Survey (USGS) Mercury Research Laboratory (Middleton, Wisconsin) via express package delivery for Hg analysis. Two Au traps were left unused per every 20 unknown traps, which were used as trip blanks to establish blank baselines.

### Chemical analysis

#### Soil and mine waste calcine

Soil and mine waste calcine samples were analyzed for the concentration of Hg using cold vapor atomic fluorescence spectrometry (CVAFS) following an aqua regia digestion as outlined in USEPA Method 1631 (USEPA 2001, 2002). Concentrations of Hg and methyl-Hg were determined by Battelle Marine Science Laboratory, Sequim, Washington, USA. Quality control/quality assurance for Hg and methyl-Hg analyses was established using standard reference materials (SRM’s), matrix and/or blank spikes, blind sample replicates, and method blanks. The SRM, IAEA-405 (estuarine sediment, certified value = 0.81 µg/g), was analyzed with the samples and recoveries of Hg varied from 99 to 104 % of the certified value. Blank spike samples were analyzed with the samples and recoveries for Hg ranged from 87 to 99 %. Matrix spikes of samples were also used and Hg recoveries ranged from 93 to 122 %. Analysis of blind sample replicates indicated a relative percent difference (RPD) of 5 to 7 % for Hg. Method blanks were below the lower limit of determination of 5 ng/g for Hg analysis of these solid samples.

Concentrations of methyl-Hg were determined in the solid samples using CVAFS following USEPA Method 1630 (USEPA 1998). To avoid possible methyl-Hg artifact effects during analysis, the samples were extracted into methylene chloride during digestion following a previously described method (Bloom et al. 1997). Recoveries of methyl-Hg varied from 78 to 86 % of the certified value for IAEA-405 (certified value = 5.49 ng/g). Recoveries of methyl-Hg matrix spikes in soil ranged from 78 to 105 %. The RPD of methyl-Hg in blind sample replicates varied from 7 to 21 %. Method blanks were below the lower limit of determination of 0.01 ng/g for methyl-Hg analysis of these solid samples.

Soil samples were also analyzed for total organic carbon (TOC) by subtracting carbonate C from total C concentrations. Total C was determined using an automated C analyzer with an infrared detector that measures CO_2_ gas liberated following sample combustion at 1,370 °C. Carbonate C was determined by liberating CO_2_ following treatment with 2 N HClO_4_, where CO_2_ was collected in a solution of monoethanolamine, which was then coulometrically titrated using platinum and silver/potassium-iodide electrodes. The RPD in stream sediment sample replicates was ≤15 % for TOC, and the lower limit of determination was 0.05 %.

#### Water

Concentrations of Hg were determined in stream, well, and spring water samples using CVAFS following EPA Method 1631 (USEPA 2002) by Battelle Marine Sciences Laboratory. Methyl-Hg was determined in the water samples following EPA Method 1630 using CVAFS (USEPA 1998). Quality control/quality assurance for Hg and methyl-Hg analyses was evaluated using matrix spikes, SRMs, sample replicates, and method blanks. Recoveries for Hg using matrix spikes in water varied from 103 to 105 % and for methyl-Hg were 92–100 %. The SRM, NIST 1641d (water solution, certified Hg value = 1.56 µg/g), was analyzed in this study, and Hg recovery was 94–96 % of the certified value. Samples of the SRM DORM-2 (dogfish muscle, certified value = 4.47 µg/g) were digested and analyzed with the water samples to assess the accuracy of the methyl-Hg method. Recoveries of methyl-Hg for DORM-2 varied from 80 to 85 % of the certified value. The RPD in water sample replicates varied from 16 to 20 % for Hg and 4–9 % for methyl-Hg. Method blanks were less than the lower limit of determination of 0.2 ng/L for Hg and 0.02 ng/L for methyl-Hg in water.

DOC was determined in the water samples collected in this study using a Shimadzu TOC-V_CSH_ instrument at the USGS, Denver, Colorado. In the water samples, organic carbon was oxidized to CO_2_ by high temperature catalytic oxidation and CO_2_ was measured by a non-dispersive infrared detector. The precision of the DOC method was ±10 %. Field and method blanks contained DOC below the lower limit of determination of 0.3 mg/L. Several on-site field measurements were also taken in water using a Hydrolab Quanta^®^ instrument, but of these only temperature, pH, dissolved oxygen, and electrical conductivity are reported here (Table 2).

**Table 2:** Geochemical data for water samples

Sample	Hg ng/L	Methyl-Hg ng/L	Methyl-Hg/Hg %	DOC mg/L	pH	DO mg/L	Conductivity μs/cm	T °C
*Mine runoff*								
LGD1	11	0.24	2.2	5.3	7.4	5.5	3,200	20
RRN1	14	0.15	1.0	5.0	7.9	4.6	2,030	27
TCK2	7.3	0.79	11	6.0	8.7	7.3	250	17
TCK3	7.9	1.2	15	6.2	8.3	6.0	280	21
*Baseline streams*								
TOR1	9.3	0.14	1.5	3.7	8.1	3.5	850	33
TOR2	8.3	0.12	1.4	3.5	7.9	5.7	900	26
TOR3	9.7	0.11	1.1	4.5	8.3	5.7	980	21
Ttrbl	4.2	0.07	1.6	2.9	7.7	4.9	1,460	21
CatFl	1.1	0.03	3.0	n.d.	7.1	5.5	400	19
TCK1	2.1	0.61	11	6.2	8.6	6.5	270	16
*Springs and wells*								
Hotl	1.5	0.03	2.1	1.1	7.3	n.d.	1,300	41
GlnSl	1.5	0.02	1.4	1.6	7.6	6.0	710	23
Mosql	1.2	<0.02	<0.83	n.d.	7.3	3.1	1,270	33
CrotSl	3.1	0.07	2.1	1.8	8.8	6.1	3,520	20
OakSl	1.1	0.02	1.9	1.1	7.4	5.3	380	16
Snaill	0.54	<0.02	<1.9	1.0	7.1	4.1	220	20
MEarl	0.05	<0.02	<20	n.d.	7.4	6.0	530	25
ButlSl	0.33	<0.02	<3.0	n.d.	7.4	4.8	420	23
*Drinking water*								
BASN1	6.9	<0.02	<0.14	1.0	7.6	5.0	390	19
CAST 1	0.74	<0.02	<1.4	1.2	7.9	5.1	510	20
RGV1	1.0	<0.02	<1.0	n.d.	7.2	1.9	1,250	35
RGVC	1.1	<0.02	<0.91	n.d.	7.3	5.3	1,120	22
BARK1	0.63	<0.02	<1.6	1.1	7.1	3.0	1,100	26
PANJ1	9.1	0.10	1.1	n.d.	7.5	4.2	390	25
*Rio Grande*								
RIO1	13	0.35	2.7	4.0	7.9	5.5	1,590	22
RIO2	6.5	0.90	14	4.8	8.0	5.5	1,140	20
RIO3	3.0	0.28	9.3	3.1	7.8	4.3	1,390	21
RIO4	2.7	0.34	13	3.7	7.8	4.7	910	22
RIO5	7.3	1.9	26	6.9	7.8	4.5	760	21
RIO5F	6.5	0.22	3.3	3.5	8.2	8.1	1,410	22
RIO6	3.2	0.51	16	3.3	7.8	5.2	770	20
RIO7	2.9	0.31	11	3.2	7.6	7.4	790	21
RIO8	4.0	0.60	15	3.2	7.6	6.8	980	22
RIO9	3.8	0.58	15	3.4	7.8	7.9	1,600	22

Sample numbers correspond to those shown in Fig. 1

#### Leachates

Concentrations of Hg were determined in water leachate samples using CVAFS at the USGS, Denver, following a previously published method (Kennedy and Crock 1987). Quality control/quality assurance for Hg analysis of these leachates was addressed through the use of several internal water laboratory standards, sample replicates, and method blanks. The RPD of Hg in sample replicates varied from 3 to 17 %. Recovery of Hg from internal laboratory standards varied from 87 to 97 %. Method blanks were less than the lower limit of determination of 5 ng/L for Hg.

#### Air and soil gas Hg

In the laboratory, Hg amalgamated to the Au beads on the sample traps was quantified by thermal desorption of the Hg at 450 °C onto an ultra-high purity Ar carrier gas, which was then detected by CVAFS using a Tekran model 2,500 Hg detector at the USGS Mercury Research Laboratory. Quality control/quality assurance was addressed using trip blank Au traps and blind sample replicates. Trip blanks indicated a lower limit of determination of 0.03 ng. The RPD Hg for blind sample replicates was 9–19 %.

## Results and discussion

### Soil

**Fig. 3:**
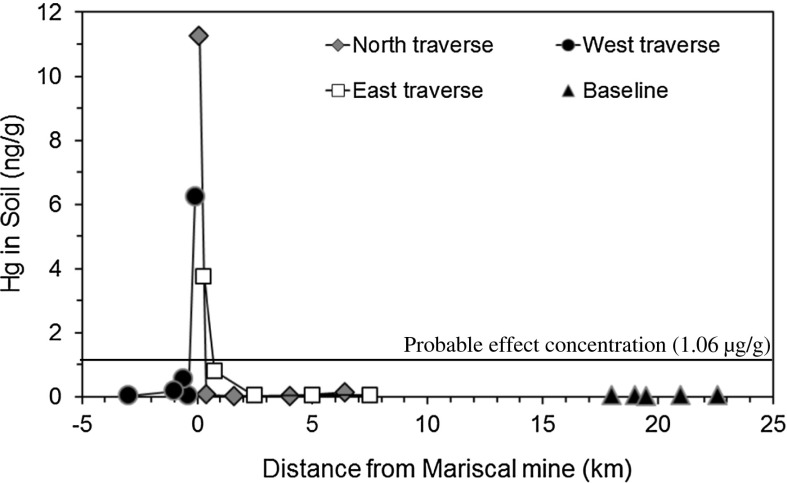
Concentration of Hg in soil versus distance from the Mariscal mine. Negative distances are those for the west traverse. The probable effect concentration for Hg (MacDonald et al. 2000) is shown for reference

Samples of A-horizon soil collected within 300 m of the Mariscal mine site contained elevated concentrations of Hg that ranged from 3.8 to 11 µg/g (Table 1, Fig. 3). Other soil samples collected more distally (>300 m, up to 7.5 km) from the Mariscal mine contained considerably lower Hg concentrations that varied from 0.02 to 0.81 µg/g (Fig. 3). Soil collected from the most distal locations along each of the three transects contained Hg that varied from 0.04 to 0.14 µg/g and was similar to Hg found in soil collected from uncontaminated baseline sites (0.03–0.05 µg/g). Only 3 of the 20 collected soil samples (those closest to the mine, Fig. 3) exceeded the probable effect concentration for Hg of 1.06 µg/g, above which harmful effects are likely to be observed in sediment-dwelling organisms (MacDonald et al. 2000). Similarly, concentrations of Hg in the three soil samples collected most proximal to the Mariscal mine exceeded the 2.3 µg/g USEPA residential soil screening level (SSL), whereas Hg in all soil samples were lower than the 31 µg/g USEPA industrial SSL for Hg (USEPA 2013). These Hg data indicate local, windblown transport of mine waste primarily in soil within about 300 m of the mine site, although there was no correlation of Hg concentration with wind direction.

**Fig. 4:**
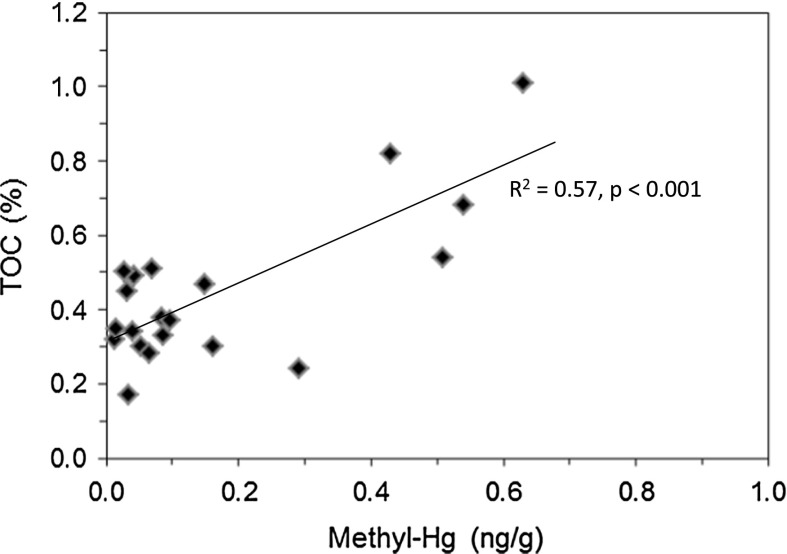
Concentration of methyl-Hg versus total organic carbon (TOC) in soil

Methyl-Hg shows a similar pattern where soil collected within 750 m of the mine contained methyl-Hg concentrations that varied from 0.04 to 0.63 ng/g, whereas soil samples collected up to 7.5 km from the mine contain methyl-Hg concentrations that varied from 0.01 to 0.29 ng/g (Table 1). Soil collected from uncontaminated baseline sites was found to contain the lowest methyl-Hg concentrations, which ranged from 0.02 to 0.04 ng/g. The ratio of methyl-Hg/Hg in soil was generally low (<1 %, Table 1), which is consistent with that found in other studies of Hg mines (Hines et al. 2000; Ullrich et al. 2001; Rimondi et al. 2012). All concentrations of methyl-Hg in the soil samples were lower than the USEPA methyl-Hg residential SSL of 0.78 µg/g and the industrial SSL of 10 μg/g (USEPA 2013). Soil samples collected in this study show a correlation between methyl-Hg and TOC (*r* ^2^ = 0.57, *p* < 0.001; Fig. 4), which is consistent with the affinity of methyl-Hg for organic matter and similar to results found in other studies of Hg mined areas (Rimondi et al. 2012; Tomiyasu et al. 2012). The methyl-Hg data for soil indicate generally low conversion of inorganic Hg (dominantly HgS) to the highly toxic methyl-Hg.

### Water

**Fig. 5:**
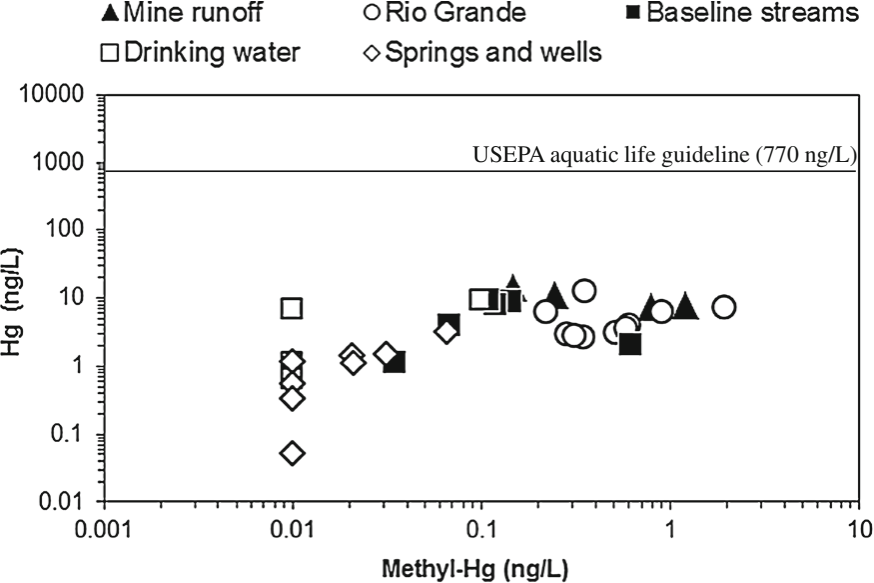
Concentration of Hg versus methyl-Hg in water. The USEPA guideline recommended to protect aquatic wildlife from chronic effects of Hg (USEPA 1995) is shown for reference

Water was collected from streams draining mined areas, streams distant from mines, the Rio Grande, hot and cold springs, wells, and drinking water supplies in and around BBNP (Fig. 1, Table 2). The highest Hg concentrations were found in mine water runoff, which varied from 7.9 to −14 ng/L (*n* = 4; Table 2). Methyl-Hg in mine runoff water ranged from 0.15 to 1.2 ng/L and was also among the highest in this study (Fig. 5). The highest Hg concentration (14 ng/L) was found in water collected from a tributary of Terlingua Creek that receives runoff from the Study Butte mine (Fig. 1, Table 2). Water collected from the Rio Grande was also elevated in Hg and methyl-Hg where Hg varied from 2.7 to 13 ng/L (*n* = 10) and methyl-Hg varied from 0.22 to 1.9 ng/L (Table 2, Fig. 5). The highest Hg concentration in Rio Grande water was collected from the site farthest upstream (RIO 1, 13 ng/L), which is upstream from mines of the Terlingua district, and thus, Hg at this site is likely from other upstream point sources in Mexico and the USA. Potential sources of Hg upstream from this site are most likely anthropogenic sources such as industrial, urban, and agricultural runoff. Lower concentrations of Hg were found in springs, wells, and drinking water supplies in BBNP, which varied from 0.05 to 9.1 ng/L (Table 2). Methyl-Hg in these water sources was the lowest in this study and varied from <0.02 to 0.10 ng/L. Baseline water samples were collected at sites distant from mines on Tornillo Creek and tributaries of Terlingua Creek, and Hg in these samples varied from 2.1 to 9.7 ng/L (*n* = 6), a variation generally similar to that of drinking water supplies in the study area. Methyl-Hg in baseline streams was generally low and varied from 0.03 to 0.14 ng/L. Dissolved oxygen in surface water samples indicate that water is generally oxidized in this environment (Table 2). The dominant form of Hg in such water is likely Hg^2+^ and methylation of Hg is generally low in oxidized environments (USEPA 1997). Similar to results for soil, the ratio of methyl-Hg/Hg was generally low (<3 %) in water samples analyzed in this study (Table 2). However, water collected from the Rio Grande was found to have methyl-Hg/Hg as high as 26 %, which was likely due to generally higher DOC in these samples (Table 2).

**Fig. 6:**
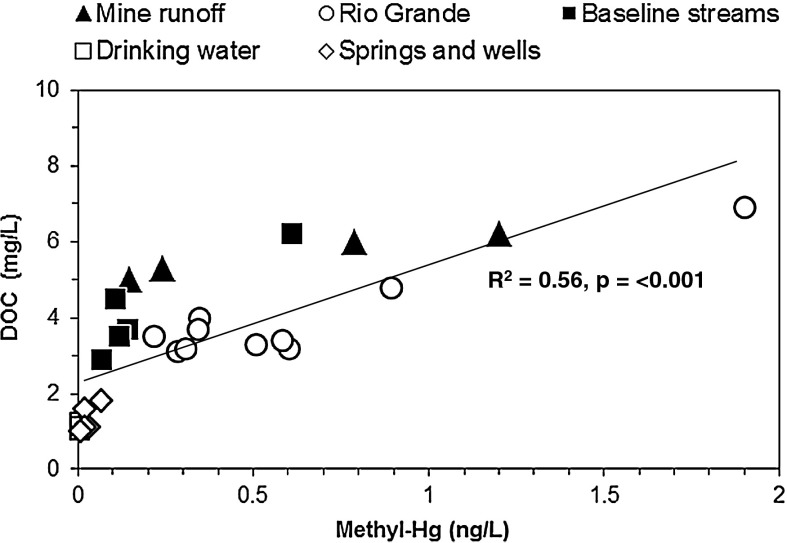
Concentration of methyl-Hg versus dissolved organic carbon (DOC) in water

Concentrations of Hg in all water samples collected in this study were considerably below established water quality guidelines for Hg including (1) the 2,000 ng/L drinking water Hg guideline recommended by the USEPA (USEPA 2009), (2) the 6,000 ng/L international drinking water Hg guideline recommended by the World Health Organization (WHO 2005), and (3) the USEPA 770 ng/L guideline recommended to protect aquatic wildlife against chronic effects of Hg (USEPA 1995). There is no recommended guideline for methyl-Hg in drinking water. However, water collected in this study indicated a significant correlation between methyl-Hg and DOC (*R* ^2^ = 0.56, *p* < 0.001; Fig. 6) suggesting that methyl-Hg has strong binding with DOC or that methylation of Hg is higher in the presence of DOC as found in other studies (Berndt and Bavin 2012; Alpers et al. 2013). A similar result was observed between methyl-Hg and TOC in soil samples.

### Leachates

Water leach studies were carried out on mine waste calcine samples collected from several localities because the study area is in an arid desert climate with little annual precipitation. Only four mine runoff water samples were collected in this study, and therefore, the USEPA 1312 synthetic precipitation leaching procedure was used to simulate potential leaching of Hg from mine waste during periods of precipitation. The obtained water leachates were filtered at 0.7, 0.45, and 0.2 µm to evaluate if Hg in the leachates was dominantly in the suspended or dissolved fraction.

**Fig. 7:**
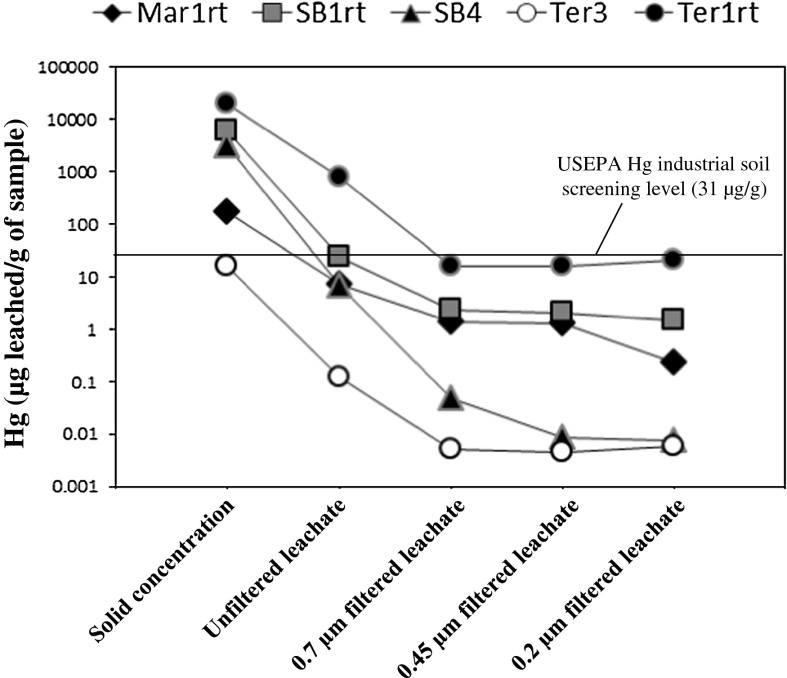
Concentration of Hg in various leachates obtained from leaching of mine waste calcine samples using the USEPA method 1312, synthetic precipitation leaching procedure (USEPA 1994). Sample numbers correspond to those in Table 3. The USEPA industrial soil screening level for Hg is shown for reference (USEPA 2013)

Concentrations of Hg in the leachates varied widely from <0.001 to 760 µg of Hg in leachate/g of sample leached (Table 3, Fig. 7). The total amount of Hg leached from the mine waste was generally low and varied from 0.02 to 4.1 % (based on Hg concentrations in the unfiltered leachates). These data are similar to, or lower than, results from other water leach studies (Gray et al. 2010). There is no recommended guideline for Hg in water leachates, so the leachate Hg concentrations (Hg in leachate/g of sample leached) were compared to the USEPA Hg industrial SSL (USEPA 2013). Concentrations of Hg in leachates were generally found to be lower than the 31 µg/g industrial SSL (USEPA 2013). Although all Hg concentrations in the mine waste calcine samples exceeded this industrial SSL, only the Hg concentration of one unfiltered leachate (Ter1rt, 760 µg/g), which was collected from inside an abandoned retort near the town of Terlingua, exceeded this SSL guideline. Concentration of Hg in leachates generally decreases with decreasing pore size of filtration indicating that Hg in the leachates is dominantly suspended particulate Hg, most likely suspended particulate cinnabar released during leaching.

**Table 3:** Concentrations of Hg for leachates of mine waste samples

Sample	Description	Mine waste Hg (μg/g)	Unfiltered μg Hg leached/g sample	0.7 nm μg Hg leached/g sample	0.45 μm μg Hg leached/g sample	0.2 μm μg Hg leached/g sample
Mar1rt	Mariposa mine, retort	170	7.0	1.4	1.3	0.23
Mar2	Mariposa mine, calcine	190	1.1	0.098	0.10	0.11
Mar3	Mariposa mine, calcine	35	0.15	0.012	0.006	0.005
Mar4	Mariposa mine, calcine	25	0.81	0.012	0.012	0.012
Mar5	Mariposa mine, calcine	13	0.13	0.005	0.011	0.006
Ter1rt	Terlingua mine, retort	19,000	760	16	16	20
Ter2	Terlingua mine, calcine	14	0.040	0.015	0.021	0.020
Ter3	Terlingua mine, calcine	16	0.12	0.005	0.005	0.006
Ter4	Terlingua mine, calcine	170	0.030	0.002	0.002	<0.001
Ter5	Terlingua mine, calcine	4.1	0.040	0.003	0.002	<0.001
MSM1	Mariscal mine, calcine	6.9	0.046	<0.001	<0.001	<0.001
MSM2	Mariscal mine, calcine	31	0.040	<0.001	<0.001	0.002
MSM3	Mariscal mine, calcine	44	0.31	<0.001	<0.001	<0.001
MSM4	Mariscal mine, calcine	110	0.66	<0.001	<0.001	<0.001
MSM5	Mariscal mine, calcine	150	0.62	<0.001	<0.001	<0.001
SB1rt	Study Butte mine, retort	5,900	24	2.4	2.0	1.5
SB2	Study Butte mine, calcine	12	0.13	0.006	0.006	0.007
SB3	Study Butte mine, calcine	480	0.61	0.002	<0.001	<0.001
SB4	Study Butte mine, congener soot	3,000	6.6	0.048	0.009	0.007
SB5	Study Butte mine, calcine	35	1.2	0.002	0.002	0.002

Sample numbers correspond to those shown in Fig. 1

### Air and soil gas Hg

**Fig. 8:**
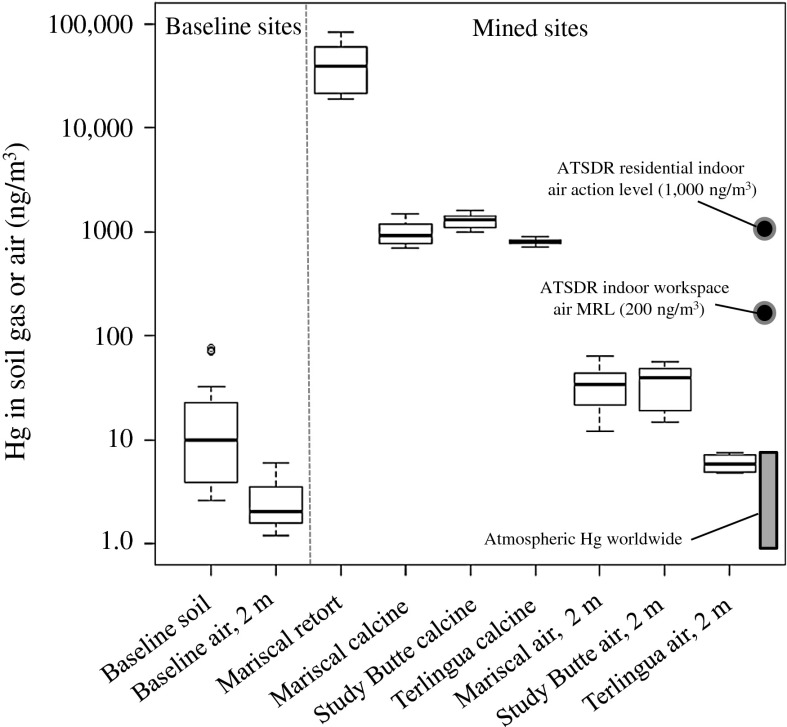
Box and whisker plot of Hg in soil gas and air samples. The *box* represents the interquartile range (IQR), where the *upper* and *lower limit* of the *box* represents the 0.75 and 0.25 quartile of the data, respectively. The *solid line* in the *box* is the median data point. The *top* and *bottom* whiskers are 1.5 X the IQR. The *open circles* (o) outside of the whiskers are outlier data exceeding 1.5 X the IQR. Points of reference for indoor air space are from the ATSDR, Agency for Toxic Substances and Disease Registry (ATSDR 2012). The range of concentration for atmospheric Hg worldwide (Fitzgerald 1986; Porcella 1994; Lamborg et al. 2002) is also shown for reference

Concentrations of Hg were measured in soil gas and air at mined and baseline sites in this study to evaluate the variability of Hg near ground surfaces in and around BBNP (Table 4). Concentration of Hg in these air and soil gas samples ranged widely from 1.2 to 82,000 ng/m^3^ (Fig. 8). The highest Hg concentrations were found in soil gas emitted from an area around an abandoned brick retort at the Mariscal mine, which varied from 19,000 to 82,000 ng/m^3^ (Table 4). Highly elevated Hg concentrations were also observed in soil gas collected from mine waste calcine piles at three mined sites, which ranged from 690 to 1,600 ng/m^3^ (Table 4). Such highly elevated Hg concentrations reflect the presence of ${\text{Hg}}_{{({\text{L}})}}^{0}$ in mine waste, which has been reported in previous studies (Kim et al. 2003; Gray et al. 2010). Conversely, concentrations of Hg measured in ambient air 2 m above the ground surface at these mined sites were found to be as much as several orders of magnitude lower, ranging from 4.9 to 64 ng/m^3^. These 2 m Hg air data suggest that although concentrations of Hg emitted from mine waste in mined areas were elevated, persistent wind in southwest Texas disperses Hg in air within a few meters of the ground surface. Other studies have observed decreasing Hg emissions from soil in mined areas over periods of hours to days (García-Sánchez et al. 2006; Higueras et al. 2012), but long-term Hg emissions from soil was not evaluated in this study. Concentrations of Hg reported in this study for air and soil gas in mined areas are similar to, or higher than, Hg measured in other areas mined for Hg, where concentrations of Hg have been reported to vary from 100 to 6,000 ng/m^3^ (Ferrara et al. 1991, 1998b).

**Table 4:** Concentration and flux of Hg in soil gas and air

Mines	*n*	Hg ng/m^3^	Hg (mean) ng/m^3^	Flux ng/m^3^/h
*Study Butte*				
Soil gas–calcine	10	1,000–1,600	1,270	1,630
Air @ 2 m	10	16–57	36	
*Mariscal*				
Soil gas–retort	10	19,000–82,000	43,000	56,700
Soil gas–calcine	12	690–1,500	990	1,260
Air @ 2 m	16	16–64	35	
*Terlingua*				
Soil gas–calcine	8	720–910	803	1,050
Air @ 2 m	8	4.9–7.6	6.1	
Baselines				
*DagF*-*b*				
Soil gas	9	3.0–17	8.9	7.9
Air @ 2 m	6	1.2–6.0	2.9	
*GVH*-*b*				
Soil gas	9	3.1–28	14	15
Air @ 2 m	6	1.2–5.0	2.5	
*MAV*-*b*				
Soil gas	9	3.0–12	6.6	5.9
Air @ 2 m	6	1.4–3.0	2.1	
*BOQ*-*b*				
Soil gas	3	2.6–4.2	3.6	2.1
Air @ 2 m	3	1.9–2.1	2.0	
*BoneS*-*b*				
Soil gas	3	25–32	28	33
Air @ 2 m	3	2.1–5.0	3.0	
*IHM*-*b*				
Soil gas	3	71–77	73	90
Air @ 2 m	4	3.1–6.0	4.5	

Mines studied and baseline sample numbers correspond to those shown in Fig. 1

Concentrations of Hg in air and soil gas from the baseline sites were among the lowest in this study. Soil gas Hg at the baseline sites varied from 2.6 to 77 ng/m^3^, whereas air collected at 2 m above the ground surface ranged from 1.2 to 6.0 ng/m^3^. However, one of the baseline sites (IHM-b, Fig. 1) was within 2 km and downwind from the Study Butte mine, this site had the highest Hg concentrations of the baseline sites and was potentially affected by windblown particulates from this mine (Fig. 1, Table 4). Concentrations of Hg measured in air at the 2 m height at the baselines sites were generally similar to that found for atmospheric Hg worldwide, which has been reported to range from 1 to 9 ng/m^3^ (Fitzgerald 1986; Porcella 1994; Lamborg et al. 2002). In this study, concentrations of Hg in all of the air collected 2 m above the ground surface were below two Hg in air guidelines recommended by the Agency for Toxic Substances and Disease Registry (ATSDR): (1) the 1,000 ng/m^3^ residential indoor air action level (remediation is needed if this level is exceeded) and (2) the 200 ng/m^3^ minimal risk level for chronic exposures of Hg (more than 365 days) for indoor workspace (ATSDR 2012). These ATSDR guidelines are for closed air spaces and thus are not directly comparable to the air and soil gas Hg data shown here. In addition, the World Health Organization recommends a “no-observed-adverse-effect” guideline of 1,000 ng/m^3^ for Hg vapor in air (WHO 2000). There are numerous homes and business buildings in and around the town of Terlingua, but concentrations of Hg were not measured in closed building air spaces in this study, and thus, any adverse effects to residents in this area are unknown. There are no homes or permanent structures at the Mariscal mine site in BBNP.

Using another approach, Hg concentrations were converted to Hg flux using:

$$F = Q \, \left( {\left( {C_{0} - C_{1} } \right)/A} \right)$$

where *F* is the flux of Hg (ng/m^2^/h), *Q* is the flow rate through the chamber (m^3^/h), *C*_o_ is the soil gas Hg concentration (ng/m^3^), C_i_ is the ambient air Hg concentration (ng/m^3^), and *A* is the area of the chamber (m^2^). Similar to the results for Hg concentrations, calculated Hg flux at the studied mined sites was found to be highly elevated ranging from 1,050 to 56,700 ng/m^2^/h (Table 4). Such highly elevated Hg flux in the Big Bend region is similar to, or higher than, that observed in other mined areas, which has been reported to be as high as 27,600 ng/m^2^/h (Engle et al. 2001; Wang et al. 2005; Eckley et al. 2010; Higueras et al. 2014). Although Hg emissions are high in such mined areas, some studies have indicated that Hg emissions from areas mined for Hg are a smaller source of Hg to the global budget of Hg compared to much larger sources such as fossil fuel burning, evaporation from oceans, and various land sources (Mason et al. 1994; Ferrara et al. 1998a; Fitzgerald et al. 1998; Lamborg et al. 2002; Kocman et al. 2013). Studies of Hg emissions at Almadén, Spain, the world’s largest Hg district from which Hg production was several orders of magnitude greater than that in the Terlingua district, indicated that Hg emissions from Almadén represented only about 0.1 % of the global anthropogenic Hg emissions (Ferrara et al. 1998a; Higueras et al. 2013). Furthermore, other studies have indicated that Hg emissions from all mined areas worldwide contribute about 1–2 % of the total atmospheric budget of Hg (Hudson et al. 1995; Kocman et al. 2013).

## Conclusions

Although concentrations of Hg in soil and water collected proximal to mines in this study were elevated, Hg in similar samples collected more distally from mines were generally lower, indicating considerable dispersion of Hg in soil and water in the region. Concentrations of Hg in all soil samples were lower than the USEPA industrial SSL for Hg. Similarly, concentrations of Hg in all water samples collected in this study were found to be below recommended drinking water and aquatic life Hg guidelines in the USA. Methyl-Hg in soil showed a significant correlation with TOC (*r* ^2^ = 0.57, *p* < 0.001), and similarly, methyl-Hg in water correlated significantly with DOC (*R* ^2^ = 0.56, *p* < 0.001), results which are consistent with the affinity of methyl-Hg for organic matter. Concentrations of Hg in leachates of mine waste were generally found to be lower than the USEPA Hg industrial SSL. Soil gas and air collected from mined areas contained Hg that was considerably higher than that found at uncontaminated baseline sites in the region. Concentrations of Hg in air collected 2 m above the ground surface at mined areas were found to be several orders of magnitude lower than Hg in soil gas collected at ground level from mine wastes. The soil gas and air Hg data indicate that persistent wind in southwest Texas disperses Hg in air within a few meters of the ground surface.

Human exposure to Hg in this region is mostly likely from long-term exposure to Hg vapor in closed spaces such as buildings and residences and/or hand-to-mouth ingestion of soil or airborne mine wastes particles containing elevated Hg. There are no homes or permanent structures on the Mariscal Hg mine in BBNP, but Mariscal is a site often visited by tourists and human exposure to Hg through inhalation of Hg-rich dust particulates is short term and should be limited as much as possible. There are numerous homes in the town of Terlingua, which are located around abandoned Hg mines. Concentrations of Hg were not measured in closed building air spaces in this study, and any adverse effects to residents in this area are unknown. However, data shown herein for Hg in soil gas and air indicate rapid dispersion of Hg in air due to dispersion by persistent wind. Proper ventilation of closed air spaces would likely reduce human exposure to Hg vapor. Human exposure to methyl-Hg, a highly toxic Hg compound, is also of concern around any Hg mine. However, concentrations of methyl-Hg in soil and water in this study were generally found to be low. The study area is in the Chihuahuan Desert, a dry and highly oxidized environment, where methyl-Hg formation is generally low.
